# Emergency 33-Week Cesarean Section With 3.4 cm Type A Aortic Dissection Repair in a Patient With Marfan Syndrome: A Multidisciplinary Approach

**DOI:** 10.7759/cureus.84648

**Published:** 2025-05-22

**Authors:** Allyson Jones, George Bcharah, Nadia Islam, Hao Pan, Sarah Smiley

**Affiliations:** 1 Obstetrics and Gynecology, Creighton University, Phoenix, USA; 2 Obstetrics and Gynecology, Mayo Clinic Alix School of Medicine, Phoenix, USA; 3 Cardiothoracic Surgery, St. Joseph’s Hospital and Medical Center, Phoenix, USA

**Keywords:** aortic dissection in pregnancy, aortic repair, high-risk pregnancy, marfan syndrome, multidisciplinary care approach, pregnancy-associated cardiovascular complications, type a aortic dissection

## Abstract

Aortic dissection (AoD) in pregnancy, particularly in women with Marfan syndrome (MFS), carries a high risk for both maternal and fetal mortality. Multidisciplinary management is essential for successful outcomes in these complex cases. We present a 26-year-old G1P0 female patient at 33 weeks gestation with chronic hypertension and MFS who developed mild symptoms of type A AoD. She underwent successful concomitant cesarean section and emergency aortic repair. Through multidisciplinary coordination of obstetrics with cardiothoracic surgery (CTS) and neonatal teams, both mother and infant recovered without major complications. This case highlights the critical need for early recognition, imaging, and multidisciplinary collaboration in pregnant patients with known risk factors of life-threatening conditions, such as AoD. Stricter screening protocols and frequent monitoring may prevent catastrophic outcomes in these high-risk patients.

## Introduction

Aortic dissection (AoD) during pregnancy is a rare but life-threatening event that poses significant risks to both the mother and fetus, occurring in approximately five to six cases per million pregnancies [1-3]. Anatomically, AoD is characterized by a tear in the intimal layer of the aorta, causing blood to flow between the aortic layers, resulting in a false lumen that can compromise blood flow to vital organs [4]. Patients with connective tissue disorders, such as Marfan syndrome (MFS), are especially at risk due to the underlying structural abnormalities of the aortic wall [2, 5]. While type A dissections are uncommon in younger patients, pregnancy-associated hemodynamic and hormonal changes, including increased stroke volume (up to 30% to 50% increase), blood pressure (BP; with systolic increases averaging 10-20 mmHg during labor), and hormonal effects on the vascular media, can precipitate dissection at lower aortic root diameters [3, 5]. Classification of AoD typically relies on anatomical criteria to guide management. The Stanford classification divides dissections into Type A (ascending aorta involvement) and Type B (descending aorta involvement distal to the left subclavian artery). The DeBakey classification further subdivides dissections based on the location and extent of involvement. Early diagnosis is often challenging due to the overlapping of symptoms with other common obstetric conditions, such as preeclampsia [5]. This case highlights the importance of prompt recognition, diagnostic imaging, and multidisciplinary surgical coordination in the successful management of an acute type A AoD in a 33-week pregnant patient with MFS.

## Case presentation

**Figure 1:**
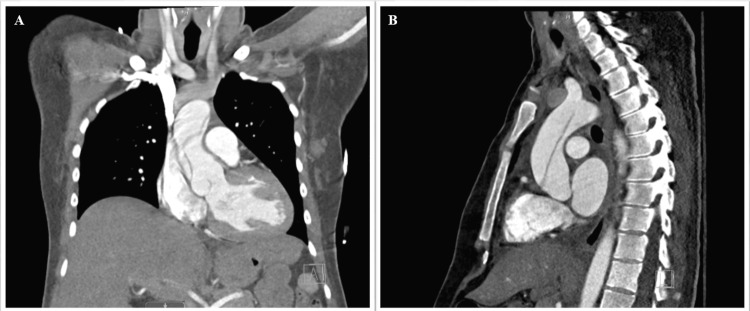
The Patient's Chest CT at Initial Presentation Showing Type A Aortic Dissection Extending to the Aortic Arch in Coronal (a) and Sagittal (B) Views.

A 26-year-old G1P0 female patient at 33 weeks gestation with a history of poorly controlled chronic hypertension and MFS with known underlying mitral valve prolapse and mild aortic root dilation (3.4 cm on a second trimester echocardiogram) presented to the emergency department with chest tightness, a dull headache, and severely elevated BPs. Despite treatment for suspected preeclampsia, her BP remained elevated. Her chest pain eventually resolved following antacid treatment and the administration of labetalol, hydralazine, and nifedipine, yet systolic levels remained in the 140s to 150s. An echocardiogram was ordered, which showed signs of a false lumen in the ascending aorta. A follow-up CT chest was performed, which confirmed a type A dissection extending from the right coronary cusp to the level of the origin of the left common carotid artery (Figures 1A, 1B).

Following multidisciplinary discussions between the obstetrics, cardiothoracic, trauma, and neonatal intensive care unit (NICU) teams, the patient was taken to the operating room. The cardiothoracic surgery (CTS) team first accessed the mediastinum via sternotomy and examined it for any effusions. The obstetrics team then performed a low transverse C-section, and a healthy preterm female infant with a low birth weight of 2,030 grams was delivered without complications. The infant’s Apgar scores were initially low, starting at one at one minute and improving to four at five minutes, six at 10 minutes, and seven at 15 minutes. The uterus was closed and demonstrated excellent tone with intravenous oxytocin. A deflated Bakri balloon was placed in the event that a postpartum hemorrhage required tamponade. The trauma team was consulted to assist with temporary abdominal packing and closure using an AbThera® vacuum-assisted closure device (3M, Saint Paul, MN).

Following the C-section, the CTS team proceeded with aortic repair. The ascending aorta had fenestrations, though the arch was intact. The ascending aorta was cross-clamped, and cardiopulmonary bypass (CPB) was initiated while cooling the patient to 26°C for circulatory arrest. Antegrade cerebral perfusion was established via the innominate artery, and the ascending aorta was transected, with the dissected portion resected down to the sinotubular junction. A 24-mm Hemashield Platinum® (Getinge, Gothenburg, Sweden) graft was anastomosed to both the proximal and distal aortic arch. The native aortic valve was intact and thus resuspended. Following cross-clamp release, spontaneous sinus rhythm resumed. Hemostasis was achieved after inspection and multiple rounds of blood product resuscitation.

Following weaning from CPB, the obstetrics team closed the abdomen. The Bakri balloon had been punctured, likely during the uterine closure after the C-section. Fundal checks post closure expelled clots greater than one liter, but the uterus remained firm with no further intervention needed.

The patient was transferred in stable condition to the ICU, was extubated within 24 hours, and subsequently transitioned to high-flow nasal cannula. She was transferred to the postnatal ward on postoperative day (POD) 3, and her chest tubes were removed on POD 4. Her BP was stabilized and monitored closely. She experienced transaminitis that self-resolved and fluid overload that required diuresis. She was discharged on POD 7 in stable condition with a multidisciplinary follow-up plan and a BP regimen of carvedilol, amlodipine, losartan, and hydralazine. The infant remained in the NICU due to prematurity but was eventually discharged in stable condition.

## Discussion

Acute AoD during pregnancy and postpartum can be fatal for both the mother and fetus [2]. Aortic root enlargement (≥4.0 cm during pregnancy), presence of a bicuspid aortic valve, and connective‐tissue disorders are associated with increased risk for type A AoDs [3]. Pregnancy in MFS is associated with an increased risk of both types A and B AoDs, with lack of knowledge of an underlying MFS diagnosis being a major contributing factor [2, 5]. Rates of AoD unrelated and related to pregnancy in women with MFS according to the GenTAC Registry are 0.6 and 5.4 per 100 patient-years, respectively [5].

Back pain (55%) or chest pain (12%) are common manifestations of AoD and can overlap with pregnancy-related symptoms, making timely diagnosis challenging [6]. The patient's chest pain and elevated BP were initially attributed to preeclampsia, which delayed the identification of the more critical AoD. Large registry analyses report that up to 6.4% of type A dissections can be asymptomatic, with multiple case reports published on painless type A dissections [7-11]. This case reinforces the importance of considering AoD in pregnant patients with MFS, especially with known risk factors (e.g., uncontrolled hypertension), even in the absence of other symptoms.

The etiology for dissection during pregnancy is multifactorial due to physiological, cardiovascular, and hormonal effects. Enhanced stroke volume and hyperdynamic circulation predispose pregnant patients to AoD, whose vascular wall integrity is already compromised from increased estrogen and progesterone [3, 12]. Dissection rates during pregnancy were found to be five times higher compared to non-pregnancy periods [13].

Current guidelines suggest monitoring pregnant patients with dilated aortic root or aortopathic conditions (e.g., MFS, Loeys-Dietz) with surveillance TTE each trimester [3, 14]. Avoiding pregnancy or attempting prophylactic aortic root replacement if the size exceeds 4 cm is also recommended [15]. Our patient's aorta dissected at 3.4 cm; thus, more frequent echocardiogram monitoring and strict control of risk factors for similar patients should be considered. Lower thresholds for intervention, increased surveillance, and larger studies are needed to guide surveillance in pregnant patients with known risk factors and aortic diameters < 4.0 cm.

Timely surgery is the treatment for acute type A AoDs, requiring more consideration in pregnant patients. Though earlier studies described cases of C-sections performed up to 16 weeks after type A dissection repair, advances in neonatal management now allow for concomitant aortic repair with the C-section [16]. Postpartum hemorrhage remains a significant risk for C-sections and increases when anticoagulation therapy is required, as was the case here [17]. The Bakri balloon was deployed to allow for a potentially needed uterine tamponade but was punctured during surgery. Alternative technologies, such as the Jada® System, which uses vacuum suction, may have offered more reliable hemorrhage control in the setting of extensive uterine manipulation required to proceed with the aortic repair [18].

## Conclusions

This case emphasizes the importance of multidisciplinary teamwork in managing complex cases and raises awareness of the increased risk of type A dissections in pregnant MFS patients with concurrent cardiovascular comorbidities. Strict screening and frequent monitoring in these cohorts is warranted.
